# Recording of fluid, beverage and water intakes at the population level in Europe

**DOI:** 10.1017/S0007114516002336

**Published:** 2016-06-21

**Authors:** Joan Gandy, Laurent Le Bellego, Jürgen König, Ana Piekarz, Gabriel Tavoularis, David R. Tennant

**Affiliations:** 1 School of Life and Medical Sciences, University of Hertfordshire, Hatfield AL10 9AB, UK; 2 British Dietetic Association, Birmingham B3 3HT, UK; 3 Danone, 91120 Palaiseau, France; 4 Department for Nutritional Sciences, University of Vienna, 1090 Vienna, Austria; 5 The Coca-Cola Company, Brussels, Belgium; 6 Centre de Recherche pour l’Étude et l’Observation des Conditions de Vie – Crédoc, 75013 Paris, France; 7 Food Chemical Risk Analysis, Brighton BN2 1FZ, UK

**Keywords:** Water intake, Dietary assessment methods, Fluid intake, Beverage intake

## Abstract

The European Food Safety Authority’s 2010 scientific opinion on dietary reference values for total water intakes was partly based on observed intakes in population groups. Large variability was observed, and it is unlikely that these differences can be explained by differences in climate, activity level and/or culture. This suggests that there are uncertainties in the methodologies used to assess water intake from food and fluids, including all types of beverages. To determine current methods for recording and reporting total water, beverages and fluid intakes, twenty-one European countries were surveyed using an electronic questionnaire. In total, twelve countries responded and ten completed surveys were summarised. Countries reported that their survey was representative of the population in terms of age and socio-economic status. However, a variety of methods were used – that is, repeated 24-h recalls, estimated food diaries and FFQ. None of the methods were validated to assess water and fluid intakes. The methods used to record liquid foods – for example, soup and diluted drinks – were inconsistent. Clarity and consistency on definitions of categories of beverages to facilitate comparisons between countries are needed. Recommendations for a unified approach to surveying and quantifying intake of water from fluids and foods are proposed.

In 2010, the European Food Safety Authority (EFSA) Panel on Dietetic Products, Nutrition and Allergies^(^ ^1^ ^)^ published their scientific opinion on dietary reference values (DRV) for total water intake. The recommended adequate intakes were based on a combination of intakes from population studies, desirable urinary osmolarity values and desirable water volumes per unit of energy consumed. The average water intakes from the population studies used by EFSA showed variability – for example, 917–1895 ml/d for women and 1027–1585 ml/d for men. The data used were taken from the EFSA Comprehensive European Food Consumption Database, which also shows high levels of variability, particularly at the lower and upper percentiles^(^ ^2^ ^)^. A similar variation was observed in an analysis of recent surveys conducted in twelve European countries, which showed that total beverage intake varied from 941 mL/d (Italy) to 2366 mL/d (Germany) for women and from 1014 mL/d (Italy) to 2659 mL/d (Germany) for men^(^ ^3^ ^)^. It is important to establish the reasons for this wide variation as it raises questions about the robustness of the observed intake data used to establish the DRV for water.

This inter-population variation in intakes is unlikely to be fully explained by climate, activity levels or culture; therefore, it is likely that it derives from the inconsistency in methodologies used. The methodologies used included 7-d weighed records, FFQ, 24-h recalls and mixed methodologies^(^ ^3^ ^–^ ^4^ ^)^. This variation was confirmed in a recent systematic review of worldwide international surveys that found that 24-h recall was the most frequently used methodology (twenty-nine out of sixty-five studies) and that twenty-two of these studies utilised single 24-h recalls^(^ ^5^ ^)^.

Water is consumed via food and fluids or beverages, which include drinking water and water in fluids such as soft drinks, coffee, tea and alcoholic beverages. There is often confusion about the terms beverages and fluids. For the purposes of this study, the term total water intake will be used to refer to water from all sources, including food; fluid intake will be used to refer to the consumption of drinking water and all other beverages and drinks.

It has been suggested that the lack of consistency in methodologies, beverage classification and measurement units may result in underestimates of total fluid intake^(^ ^6^ ^)^. The type of methodology has been shown to affect the result of intake estimates in population surveys^(^ ^7^ ^)^. A comparison of drinking water intake in two cohorts of the What We Eat in America/National Health and Nutrition Examination Survey showed significant differences in water intake between the 2005/2006 survey, which used the automated multiple-pass method for 24-h recall, and the 2003/2004 survey, which used post-recall food frequency-type questions^(^ ^8^ ^)^. However, it is important to consider that, although comparable to some extent, there still were variations in the two populations. Recently, a cross-over study reported significant differences in the estimation of total fluid intake when a 24-h recall or a 7-d fluid-specific diary was used. Although there was a significant correlation between the methods, a significant underestimate of 382 ml was observed when a 24-h recall was used. This difference increased with increasing total fluid intake^(^ ^7^ ^)^.

Population surveys use methodologies that have been validated for energy (such as the UK National Diet and Nutrition Survey (NDNS)) or particular nutrients, and there is an assumption that they are also valid for water and fluid intakes; it is probable that this assumption is invalid^(^ ^9^ ^)^. Food intake is usually structured around meals with snacks between meals, unlike fluids, which are consumed throughout the day^(^ ^10^ ^)^. This suggests that total water and fluid intakes may be underestimated, and current recommendations based on intake studies may also underestimate the population needs. With increasing interest in the impact of the quantity and composition of beverage intake^(^ ^11^ ^)^, it is important to be able to accurately assess what populations are drinking to understand possible health risks and/or benefits as well as develop future recommendations for total water intake and types of fluid.

The present study aimed to determine current methodologies used to assess water and fluid intakes across Europe by conducting a survey of twenty-one European countries. On the basis of the results of this assessment, recommendations to improve consistency were developed.

## Methods

An expert group was convened by the International Life Sciences Institute (ILSI) Europe as part of their Food Intake Methodology Task Force to investigate methodologies for recording beverage and water intakes at the population level. A questionnaire was developed to collect information on the methodologies used throughout Europe to assess water and fluid intakes in populations, including analysis and reporting (the questionnaire is available in the online Supplementary Appendix S1).

Lead scientists were identified for twenty-one countries from publications and personal contacts and were invited to complete the questionnaire electronically; twelve countries responded. Denmark, Finland, France, Greece, Norway, Poland, Portugal, Slovakia and Switzerland did not respond possibly because the correct contact person had not been identified or because of changes in contact information. One country was excluded as the survey reported food purchases rather than population intakes (Spain). Another country was excluded, as the questionnaire was incomplete (Italy). Therefore, ten questionnaires were collated and summarised.

## Results

Table 1 shows the size of the population surveys, age groups surveyed and the timing of the survey. All respondents described methods for selecting a representative sample and, if necessary, weighting the sample accordingly. Surveys were considered representative of socio-economic class; however, one respondent reported that the low-income population might not have been represented.

**Table 1:** Characteristics of population diet/nutrition surveys for ten European countries

Countries	Average population size	Age groups (years)	Timing of survey	Methodology	Method used to estimate volumes	Daily variation considered	Ensure volume consumed (rather than served) is recorded	Water content of foods calculated
Austria^(^ ^34^ ^)^	1002	7–14 and 18–80	August–February	7–14 years – 3 d dietary record, FFQ; 18–80 years – repeated 24-h recall, FFQ	Photographs	No	Yes	Yes
Belgium^(^ ^35^ ^)^	3200	2004: 18–99; 2014: 3–64	All seasons	Children – food diary*, FFQ; adolescents 24-h recall*, FFQ; adults 24-h recall* FFQ	Photographs	No	No	No
Czech Republic^(^ ^36^ ^)^	2590	4–90	All seasons	Repeated (×2) 24-h recall*	Photographs and household measures	No	No	No
Germany^(^ ^37^ ^)^	19 329	14–80	All seasons	3 dietary assessment methods applied separately 2×24 h recalls*; diet history interviews*; dietary weighing records*	Household measurements and standard units (e. g. bottles)	No	No	No
Hungary^(^ ^38^ ^,^ ^39^ ^)^	Approximately 5000	0–101	February–June	FFQ	Photographs	No	No	Yes
Iceland^(^ ^40^ ^)^	2000	18–80	All seasons	Repeated (×2) 24-h recall* – with questions on frequency of consumption of certain foods and food groups (paper version used for frequency questions)	Pictures or standard glass 200 ml, standard cup 150 ml	No	Yes	Yes
The Netherlands^(^ ^41^ ^)^	3819	Each survey targets a defined population for example 2007–2010: 7–69 years	All seasons	Repeated (×2) 24-h recall*	Choice of – photographs, household measures, weight, volume or standard units	No	Yes	Yes
Republic of Ireland^(^ ^42^ ^)^	500	Each survey targets a defined population for example 2005–2006: 13–17 years; 2008–2010: 18–90 years	All seasons	4 d weighed food diary	Weighed intake Otherwise photographs or household measures	Yes	Yes	Yes
Sweden^(^ ^43^ ^)^	1200	18–74 years	All seasons	7 d estimated food diary	No response	Yes	No	No
UK^(^ ^32^ ^)^	6828 1–4 years Every year the study aims to recruit 1000 core participants 500 adults and 500 children	18 months upwards	All seasons – rolling programme	4 d estimated food diary	Respondents are given an image of a glass and examples of volume sizes in a table. Usual cup/mug volume measured	Yes	Yes	No

* Electronic versions used.

### Methodology

Table 1 shows the methodologies used by the ten European countries. All but one respondent stated that all drinking events were captured. Only countries using food diaries considered variation across days of the week. None of the respondents reported using methodologies that were validated for the assessment of water and fluid intakes. Results from the survey suggest that efforts have been made to quantify fluid and water intakes as shown in Table 1. However, 50 % of the countries asked participants to record the quantity served rather than the quantity consumed.

There was a lack of consistency on how to report foods with high liquid content, for example, soups; two countries asked for ingredients of such food, whereas six countries recorded type and quantity analysing them as composite dishes, although there was no indication of whether or not water and/or stock used in these foods were recorded. Half of the countries reported calculating water content of foods but only the Republic of Ireland reported a value of 33 % of water from food^(^ ^12^ ^)^, which is higher than the range (20–30 %) used by EFSA^(^ ^1^ ^)^.

Data collection on dilution of cordials, powders and concentrates was inconsistent. Four countries recorded the volume of prepared drinks, and three recorded products and water separately; one country used a standard dilution factor and another used dilution factors based on age. The reporting and analysis of such beverage require further investigation and clarification.

### Water sources

All respondents reported collecting information on the type of water consumed – that is, tap or bottled water – with all but one categorising further into still or sparkling bottled water. At least one code for water was used (other codes being used for type of water) in the food composition databases.

### Categorisation of beverages

The categories used by respondents in the present survey were consistent for water, milk and milk product categories; eight respondents split soft drinks into four categories – namely, still regular and low-energy (diet) and carbonated regular and low-energy (diet) drinks. All but one included alcoholic beverages as a separate category, and five included energy drinks in a separate category.

### Analysis and reporting

In all, four countries reported cleaning data, with two using energy intake to identify and correct unreliable records; one country used the Goldberg’s cut-off as described by Black^(^ ^13^ ^)^, and two countries noted extreme values but did not exclude these data.

## Discussion

This study reports current practices for assessing fluid and water intakes in populations in a sample of European countries. The results of this survey suggest that there is growing recognition of the importance of hydration and the types of beverages consumed on health. However, concerns about the methodological variation and validity of the surveys were raised by the responses.

The present study confirms the variation in methodologies previously shown^(^ ^3^ ^–^ ^5^ ^)^. The development of a unified approach to dietary assessment, particularly assessment of water and fluid intakes, would facilitate comparisons between countries^(^ ^14^ ^–^ ^16^ ^)^ and the development of recommendations. EFSA^(^ ^17^ ^)^ recently published guidance on producing high-quality consumption data that is harmonised throughout Europe^(^ ^18^ ^)^. EFSA recommends recording data on 2 non-consecutive days using 24-h dietary recalls by a computer-assisted personal or telephone interview (CAPI/CATI). In infants and children, the recommendation is to use two 24-h food diaries followed by CAPI/CATI. Further, the use of software programmes including automatic checks/pathways and questions is recommended to ensure the inclusion of foods that are easily forgotten such as between-meals drinks. An international panel on water quality has reviewed the literature on assessment of water intake and exposure studies and recommended the use of a 4-d diary^(^ ^19^ ^)^; repeated 24-h recall was the second method of choice. At present, there is no consensus regarding dietary assessment methodology to be used in studies on health and well-being, and the need to have validated methodology still remains.

The results of the present survey highlight some of the limitations of using a methodology that is not designed to assess water and fluid intakes. Although most respondents reported capturing all drinking events, it is likely that the current methodologies emphasise consumption during meal times and not throughout the day, suggesting that some events may not be recorded^(^ ^20^ ^)^. In addition, the type of beverage or drink may vary throughout the day. For example, alcohol is more likely to be consumed in the evening^(^ ^10^ ^)^. People often drink small volumes of water throughout the day and find this difficult to quantify. A limitation of the questionnaire is that questions about sipping from bottles, bought or home filled, or drinking from fountains were not included. The addition of a meal occasion ‘during the whole day’ may facilitate recording of this volume.

Although the methodologies may be validated for energy and other nutrients – for example, in the UK’s NDNS^(^ ^21^ ^)^, none reported that the methodology was validated for water and fluid intakes. It is vital that methodologies are validated in order to identify and establish dose–response relationships between water and fluid intakes and disease^(^ ^22^ ^)^, as well as to develop robust recommendations.

It is reassuring that most countries sample across the year as seasonal variations in diet will introduce possible errors in recording water and fluid intakes^(^ ^23^ ^)^, a concern that may not be present to the same extent as for other nutrients. However, only countries using methods that capture >1 d of consumption considered variation across days of the week. Individuals do not eat or drink the same items each day, and failure to consider this in the methodology introduces substantial errors^(^ ^24^ ^,^ ^25^ ^)^. For instance, fluid consumption has been shown to be higher during the weekend (Friday–Sunday) for men and higher for women on Friday and Saturday^(^ ^10^ ^)^. In addition, differences in the volume of some types of fluid consumed across the week have been reported. It is probable that population surveys do not capture this daily variation, and are therefore underestimating water and fluid consumption.

Results from the survey suggest that efforts are made to quantify fluids with photographs and/or sample cups and glasses. However, it is a concern that only half of the countries asked participants to record quantity consumed rather than recording the quantity served. This is an issue that needs to be addressed by developing clear guidelines that reflect the intake patterns of fluid consumption, and subsequently training participants on how to record intakes.

There was a lack of clarity and consistency on how to report foods with high liquid content, for example, soups. More information on preparation and the inclusion of water and/or stock should be recorded. Treating these foods as composite foods or using recognised food categories such as those in FoodEx2^(^ ^18^ ^)^ would ensure that water intake from these foods is recorded more accurately. Furthermore, the water content of foods should be calculated and published. EFSA^(^ ^1^ ^)^ estimates that the water content in European foods is 20–30 %; however, this figure will vary between countries and seasons depending on dietary patterns and food-types consumed – for example, it has been estimated to be 19 % in the USA^(^ ^26^ ^)^, 33 % in the Republic of Ireland^(^ ^12^ ^)^ and 40 % in China^(^ ^27^ ^)^. Accurate estimates of water content of food in the diet from more countries will aid the production of future recommendations. The recording of fluids that are diluted – for example, cordial, concentrate and squash – was varied with no consistency in dilution factors or details recorded. The only country that has clear guidelines on diluents is the UK^(^ ^28^ ^)^, which now records dilution water separately.

All respondents reported collecting information on the type of water consumed – that is, tap or bottled water – and were coded accordingly. This was a significant change from previous studies^(^ ^29^ ^)^, suggesting an increased interest in water and fluid consumption and its relation to health. With rising interest in the type of fluids or beverages consumed and health^(^ ^11^ ^,^ ^30^ ^)^, it is vital that beverages are categorised consistently. It appeared that there was some uniformity of categories across the countries surveyed; however, definitions of the categories for each country were not collected, and it is possible that there may be some confusion and overlap. Consistent, uniform definitions of the categories would facilitate between-country comparisons and collation of pan European data. Adoption of EFSA’s^(^ ^17^ ^)^ recommendation that food and beverage intake be reported according to their FoodEx2^(^ ^18^ ^)^, food classification system in the EU Menu project may be a way of dealing with this issue. In addition, participants may be confused about what category a particular fluid should be placed in. Clear definitions of categories and appropriate training for participants and survey personnel would facilitate correct and comparable categorisation.

Biomarkers are increasingly being used in population studies^(^ ^31^ ^–^ ^33^ ^)^, and their use would further elucidate the relationship between water intake and hydration status, especially in habitual low and high drinkers in populations. This would facilitate the investigation of dose–responses of water or other fluids and health status and/or specific disease – for example, renal disease and water intake. However, this would increase the cost of the survey and would require ethics approval, factors that require careful consideration. None of the countries reported using hydration.

The assessment of fluid and water intakes is complex and presents significant challenges. The results of this survey show that there is increasing awareness of the need to accurately record water, fluids and liquid food intake in population dietary surveys. However, there is still a need to develop a consistent and validated methodology to avoid ambiguity and reduce potential sources of error. Development of a unified approach to the assessment of water and fluid intakes across Europe would facilitate valuable insights into the relationship between hydration and fluid type and health. It is vital that methodologies are validated for fluid and water intakes to enable dose–response comparisons to be made and to provide more robust data for future recommendations.

## Supplementary material

For supplementary material accompanying this paper visit http://dx.doi.org/10.1017/S0007114516002336. click here to view supplementary material
