# Supplementary material for: Recording of fluid, beverage and water intakes at the population level in Europe
Source: Br J Nutr. 2016 Jun 21;116(4):677–82. doi: 10.1017/S0007114516002336 (PMC4970442; doi:10.1017/S0007114516002336)
Supplement: Supplementary file 1 [file S0007114516002336sup001.pdf]

ILSI Europe
International Life Sciences Institute

FC/52/JN14

ILSI Europe A.I.S.B.L.
83 Avenue E. Mounier, box 6
B-1200 Brussels
Belgium

Tel : +32-2 771.00.14
Fax : +32-2 762.00.44
info@ilsieurope.be
www.ilsi.eu

FOOD INTAKE METHODOLOGY TASK FORCE EXPERT GROUP ON ‘ADEQUATE METHODOLOGIES FOR RECORDING FLUID AND WATER INTAKE AT POPULATION LEVEL’

QUESTIONNAIRE

Thank you for taking part in this survey.

Fluid and water intake have received limited attention in national food surveys. Comparison of existing surveys suggests that the methodology impacts tremendously the reported quantities of fluid drunk, particularly water. Many of these surveys are conducted using methodologies validated to record food intake and do not necessarily consider the intricacies of fluid consumption in their design. This questionnaire is designed to provide a better understanding the methods currently used to assess population water/fluid consumption.

The first part of the questionnaire asks about general information, description of your national food/diet survey and sample details. The second part aims to gather information on the analysis and reporting of the survey.

Filling in the questionnaire takes about 15-20 minutes.

Part 1
General information and description of the survey

Which country and/or region does your survey represent?

What is the name of your national survey?

Who are the owners of the data (e.g. Government organisation, university, survey company)?

How frequent is your national dietary survey?
- Annual
- Every other year
- Every 3 years
- Every 4 years
- Every 5 years
- A rolling programme
- Other – please specify

Please describe any governance processes for the survey e.g. data protection, ethical or other approvals.

Survey sample

How is the sample recruited? e.g. Advertisements in national or local press, through health centres, on line media, by a sampling quota method. Please give brief details.

How representative is the survey of the national population e.g. age, gender, social/economic status, ethnicity, regional distribution?

Does the survey cover all socioeconomic classes?

How is socioeconomic class defined e.g. national classification?

How do you ensure that the sample is representative?

What type of sampling was used?
- Random
- Simple random sampling
- Stratified
- Cluster
- Systematic

Non random
- Convenience/accidental sampling
- Purposive/judgment sampling
- Quota sampling

Comments

What age groups are surveyed?

What time of the year are surveys conducted?

What is the average size of the study sample?

Survey methodology

What dietary assessment methodology is used?
- 24 hour recall
    - If repeated please give frequency and period between data collections.
- Estimated food diary
    - Number of days surveyed
    - Days of the week surveyed
- Weighed food intake
    - Number of days surveyed

- Food frequency questionnaire (FFQ)
    - How many items are included in the FFQ?
- Other method e.g. a combination of methods

    Please give details

Do you use electronic or paper diaries?

Please give details.

Has the survey methodology been published?

If so please give brief details.

What food description/coding system is used?

What other biometric data (age, bodyweight, etc.) are collected?

Is each survey conducted at the same time of the year?

Please give details.

How do you check for completeness of the survey questionnaires e.g. follow up phone calls?

<u>Fluid intake</u>

How does the survey capture drinking acts?

Does the survey capture drinking acts between/outside meals?

If so, how does it do this?

Are all types of fluid reported or only specified fluids e.g. milk, juices?

How do you record liquid foods e.g. soup?

How are participants taught to estimate volumes e.g. using glasses of set volumes, photographs?

How are volumes of the fluids recorded e.g. glasses, ml?

If used please give details of the glasses/cups sizes used

Is fluid expressed as a volume or by water content?

If cordials/concentrates/powders are consumed how are dilution factors expressed?

How is this recorded? Is it recorded as just the product, the product + water, or the prepared drink?

Is additional information about the drink (e.g. low calorie, brand, flavour) recorded?

Is the type and sources of water recorded (e.g. tap, still bottled, sparkling bottled)?

Do you ensure volume drunk is recorded rather than volume served?
If so, how do you do this?

Part 2

Analysis of the fluid intake data of the survey

What was the response rate to the last survey?

What nutrient analysis (food composition) database is used? If yes, please provide a reference

Is there a code for water in the database?

Is this categorised further e.g. still or sparkling, bottled, tap water?

What categories do you have for fluids?

- Water
- Flavoured water
- Milk and milk products
- Hot beverages
- Still sweetened drinks
    - Regular
    - Diet/low calorie
- Carbonated sweetened drinks
    - Regular
    - Diet/low calorie
- Functional drinks
- Alcohol
- Others (please give details)

Do you record added items e.g. milk, sugar, lemon, concentrated fruit syrup?

Do you calculate intake of water from food?
If so, how do you do this calculation?
If so, what is the percentage of water in food?

Is the data edited/censored/cleaned in any way (e.g. mean fluid intake < 500 ml/day excluded from analysis)?

Do you collect any clinical data on hydration markers (e.g. urine osmolality)?

Do you make adjustments to the volume of water reported if the reported volume is zero or very low?

Survey reporting

Is a report of the survey available on a public website?
Yes No
If yes, please give the web site address.

Are the results published in a peer reviewed journal?
Yes No
If yes, please give details.

Are the raw data from the survey available to download?
Yes No
If yes, please give the web site address.

Do you have any further comments?

If you are not the person that was emailed initially and would like to hear about the results and resulting publication please give your name and email address.

Are any other nutrition surveys conducted in your country? If so please give details of the main contact and the name of the survey.

Thank you for taking the time to complete this survey.
